# Dioscin-induced autophagy mitigates cell apoptosis through modulation of PI3K/Akt and ERK and JNK signaling pathways in human lung cancer cell lines

**DOI:** 10.1007/s00204-013-1047-z

**Published:** 2013-04-04

**Authors:** Ming-Ju Hsieh, Te-Lung Tsai, Yih-Shou Hsieh, Chau-Jong Wang, Hui-Ling Chiou

**Affiliations:** 1 School of Medical Laboratory and Biotechnology, Chung Shan Medical University, 110, Section 1 Chien-Kuo N. Road, Taichung, 402 Taiwan, ROC; 2 Institute of Biochemistry and Biotechnology, Chung Shan Medical University, Taichung, 402 Taiwan, ROC; 3 Department of Pathology & Laboratory Medicine, MacKay Memorial Hospital Hsinchu Branch, Hsinchu, 690 Taiwan, ROC; 4 Department of Biochemistry, Chung Shan Medical University, Taichung, 402 Taiwan, ROC; 5 Department of Clinical Laboratory, Chung Shan Medical University, Taichung, 402 Taiwan, ROC; 6 Department of Medical Research, Chung Shan Medical University Hospital, Taichung, 402 Taiwan, ROC

**Keywords:** Lung cancer, Dioscin, Apoptosis, Autophagy, ERK1/2, LC3, Beclin-1

## Abstract

Our previous study has revealed that dioscin, a compound with anti-inflammatory, lipid-lowering, anticancer and hepatoprotective effects, may induce autophagy in hepatoma cells. Autophagy is a lysosomal degradation pathway that is essential for cell survival and tissue homeostasis. In this study, the role of autophagy and related signaling pathways during dioscin-induced apoptosis in human lung cancer cells was investigated. Results from 4′-6-diamidino-2-phenylindole and annexin-V/PI double-staining assay showed that caspase-3- and caspase-8-dependent, and dose-dependent apoptoses were detected after a 24-h dioscin treatment. Meanwhile, autophagy was detected as early as 12 h after an exposure to low-dose dioscin, as indicated by an up-regulated expression of LC3-II and beclin-1 proteins. Blockade of autophagy with bafilomycin A1 or 3-methyladenine sensitized the A549 and H1299 cells to apoptosis. Treatment of A549 and H1299 cells with dioscin caused a dose-dependent increase in ERK1/2 and JNK1/2 activity, accompanied with a decreased PI3K expression and decreased phosphorylation of Akt and mTOR. Taken together, this study demonstrated for the first time that autophagy occurred earlier than apoptosis during dioscin-induced human lung cancer cell line apoptosis. Dioscin-induced autophagy via ERK1/2 and JNK1/2 pathways may provide a protective mechanism for cell survival against dioscin-induced apoptosis to act as a cytoprotective reaction.

## Introduction

Dioscin, plant steroidal saponin, abundantly exists in some medicinal plants, such as *Dioscorea nipponica Makino* and *Dioscorea zingiberensis Wright,* has been widely used as an important raw material for the synthesis of steroid hormone drugs (Brautbar and Williams 2002). Previous researches have demonstrated that this compound has anti-inflammatory, lipid-lowering, anticancer and hepatoprotective effects (Wang et al. 2007a, b; Sautour et al. 2004; Kaskiw et al. 2009; Lu et al. 2011). Several previous reports revealed that dioscin was able to induce apoptosis in various carcinoma cell lines (Lin et al. 2011; Choi et al. 2010; Yu et al. 2003). Dioscin may induce apoptosis via inhibition of Bcl-2 and activation of caspase-9 and caspase-3 in Hela cells (Cai et al. 2002), as well as generation of reactive oxygen species (ROS) and apoptosis in HL-60 cells (Wang et al. 2007a, b). Furthermore, it could also significantly inhibit P-glycoprotein expression, as a potent multidrug resistance reversal agent and decrease the resistance degree of HepG2/adriamycin cells (Sun et al. 2011). However, the effect of dioscin on the autophagy-related events of human lung cancer cells has not been clearly clarified.

As a major intracellular degradation mechanism, autophagy is activated under stress conditions to promote survival during starvation or lead to programmed cell death type II under specific conditions such as the inhibition of apoptosis (Ivanov et al. 2007; da Rocha et al. 2001; Chua and Choo 2011). Autophagy is initiated by the engulfment of large sections of cytoplasm by a crescent-shaped phagophore to form autophagosomes, which then undergo acidification after maturation to become acidic vesicular organelles (AVOs) (Liu and Lenardo 2007). The final fusion of these autophagosomes with lysosomes leads to their maturation into autophagolysosomes followed by the digestion of their components by lysosomal hydrolases (Kanzawa et al. 2003; Chang et al. 2007; Gorka et al. 2005). It is now believed that autophagy has broader importance in regulating growth and maintaining homeostasis in multicellular organisms. Defective autophagy contributes to pathogenesis of a number of diseases, including myopathies, neurodegenerative diseases and some forms of cancers (Kelekar 2005). Several reports showed that an induction of autophagy appears to facilitate therapy-induced killing of tumor cells (Longo et al. 2008; Ko et al. 2009). For example, temozolomide, a pro-autophagic drug, has proven to be a promising candidate for selective killing of apoptosis-resistant glioblastomas (Kanzawa et al. 2004). The aim of the present study was to characterize the effects of dioscin and further determine the molecular mechanism cross talk between autophagy and apoptosis in dioscin-induced cytotoxicity.

## Materials and methods

### Chemicals

Dioscin of over 98 % purity was purchased from China Langchem INC. (St. Caliun, Shanghai). Stock solution of dioscin was made at 10 mM concentration in dimethyl sulfoxide (DMSO) (Sigma, St. Louis Co.) and stored at −20 °C. The final concentration of DMSO for all treatments was less than 0.1 %. 3-(4,5-dimethylthiazol-2-y1)-2,5-diphenyltetrazolium bromide (MTT), bafilomycin A1 (BafA1), 4′-6-diamidino-2-phenylindole (DAPI) and 3-methyladenine (3-MA) were obtained from Sigma Chemical Co. (St. Louis, MO, USA). General caspase inhibitor Z-VAD-FMK was purchased from Promega (Madison, WI, USA). Specific inhibitors for caspase-3 (Z-DEVE-FMK), caspase-8 (Z-IETD-FMK) or caspase-9 (Z-LEHO-FMK) were purchased from BioVision (Mountain View, CA).

### Cell culture

A549 and H1299, human nonsmall cell lung cancer cell line, obtained from the Food Industry Research and Development Institute (Hsinchu, Taiwan), were cultured in Dulbecco’s modified Eagle’s medium (DMEM) (Gibco BRL, Grand Island, NY, USA) supplemented with 10 % fetal bovine serum (FBS), 1 mM glutamine, 1 % penicillin/streptomycin, 1.5 g/l sodium bicarbonate and 1 mM sodium pyruvate (Sigma, St. Louis, Mo, USA) and maintained at 37 °C in a humidified atmosphere of 5 % CO_2_.

### Cell cytotoxicity assay

The effect of dioscin on cell growth was assayed by the MTT (3-(4,5-dimethylthiazol-2-yl)-2,5-diphenyl tetrazolium bromide) method, as previously described (Ho et al. 2011). Briefly, cells were cultured in 24-well plates (5 × 10^4^/well) and stimulated with different concentrations of dioscin (0, 1.25, 2.5 and 5 μM) in culture media. After 24 or 48 h of dioscin stimulation, MTT was added to each well (0.5 mg/ml final concentration) with a further incubation for 4 h. The viable cell number was directly proportional to the production of formazan following the solubilization with isopropanol. The color intensity was measured at 570 nm. Each condition was performed in triplicate, and data were obtained from at least three separate experiments.

### DAPI (2-(4-Amidinophenyl)-6-indolecarbamidine dihydrochloride) staining

Cells (4 × 10^5^/well) were grown on 6-well cell culture dish overnight. After being subjected to indicate treatment, cells were fixed with 2 % paraformaldehyde (Sigma, USA) for 20 min and then incubated with 0.5 % Triton X-100 (Sigma, USA) for 10 min. Extensive PBS washing was conducted between each reaction to remove any residual solvent. Cells were subjected to DAPI staining for 10 min and then observed under fluorescence microscopy equipped with filters for UV.

### Cell cycle analysis

To determine the effect of dioscin on cell cycle, cells (5 × 10^5^/ml) were first cultured in serum-free medium for starvation at 18 h and then exposed to dioscin for 24 h. Then cells were washed, fixed with 70 % ethanol, incubated for 30 min in the dark at room temperature with propidium iodide (PI) buffer (4 μg/ml PI, 1 % Triton X-100, 0.5 mg/ml RNase A in PBS) and then filtered through a 40-μm nylon filter (Falcon, USA). The cell cycle distribution was analyzed for 10,000 collected cells by a FACS Vantage flow cytometer that uses the CellQuest acquisition and analysis program (Becton Dickinson FACSCalibur).

### Annexin-V/PI double-staining

To detect apoptosis in human lung cancer cells after exposure to dioscin, an FITC annexin-V Apoptosis Detection Kit I (BD Biosciences, USA) was used to quantify cell number in different stages of cell death (Casciola-Rosen et al. 1996). Briefly, 1 × 10^5^ cells were resuspended in 100 μl 1× binding buffer (0.01 M Hepes/NaOH (pH 7.4), 0.14 M NaCl, 2.5 mM CaCl_2_). After the addition of FITC annexin-V and PI (5 μl each), the cell suspension was gently vortexed and incubated for 15 min at room temperature in the dark. Four hundred microliters of 1× binding buffer was added to each tube and analyzed by flow cytometry within 1 h.

### Quantification of acidic vesicular organelle (AVO) formation

The occurrence of AVOs was assessed by a previously described method (Zhan et al. 2012). Briefly, dioscin-treated cells were washed with PBS, followed by staining with 1 μg/ml acridine orange (diluted in PBS containing 5 % FBS; Sigma) for 15 min. Afterward, cells were washed with PBS. For quantification of AVOs, acridine orange-stained cells were harvested, washed twice with PBS, resuspended in PBS containing 5 % FBS and then analyzed by flow cytometry.

### Western blot analysis

Cell lysates were separated in a 10 or 15 % polyacrylamide gel and transferred onto a PVDF membrane (Millipore Corporation, Milford, MA, USA). The blot was subsequently incubated with 3 % nonfat milk in PBS for 1 h to block nonspecific binding and probed with a corresponding antibody against a specific protein (antibodies for Bcl-2, beclin-1, phospho-mTOR and mTOR were from Cell Signaling; anti-LC3 was from NOVUS; anti-caspase-3 was from Invitrogen; antibodies for PARP, caspase-8 and caspase-9 were from Santa Cruz; antibodies for PI3K, Akt, phospho-Akt, p38, JNK1/2 and β-actin were from BD Biosciences; antibodies for ERK1/2, phospho-ERK1/2, phospho-p38 and phospho-JNK were from Millipore Corporation) for 37 °C at 2 h or overnight at 4 °C and then with an appropriate peroxidase-conjugated secondary antibody for 1 h. After the final washing, signal was developed by ECL detection system, and relative photographic density was quantitated by a gel documentation and analysis (AlphaImager 2000, Alpha Innotech Corporation, San Lean 189 dro, CA, USA).

### In situ immunofluorescence assay

Cells were seeded into 6-well dish at a density of 4 × 10^5^ cells per dish and treated with dioscin of an indicated concentration for 12 h. After the incubation, cells were fixed with 2 % paraformaldehyde for 20 min and then incubated with 0.5 % Triton X-100 for 10 min. PBS washing was conducted between each reaction to remove any residual solvent. Afterward, fixed cells were incubated with 4 % BSA at room temperature for 2 h and then with the appropriate primary antibodies at 4 °C overnight. After overnight incubation, cells were washed and then incubated with Alexa Fluor 488-conjugated affinipure goat anti-rabbit IgG secondary antibody (Jackson Immuno Research, West Grove, PA, USA) with light protection. Meanwhile, another set of cells was subjected to DAPI staining for 10 min without antibody reaction. At the end of incubation, cells were observed under fluorescence microscopy equipped with filters for UV and Blue 488 nm.

### Statistical analysis

Statistical significances of differences throughout this study were analyzed by one-way ANOVA test. A *P* value < 0.05 was considered to be statistically significant. Values represent the mean ± standard deviation, and the experiments were repeated three times.

## Results

### Cytotoxic effects and morphological features of dioscin-treated human lung cancer cell lines

**Fig. 1:**
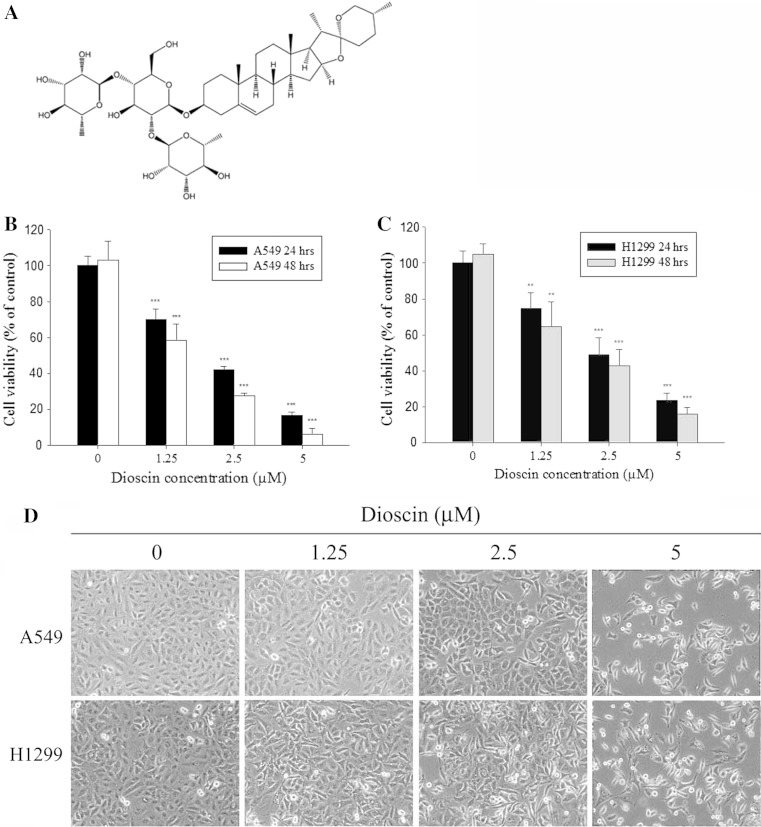
Cytotoxic effect of dioscin in human lung cancer cell lines. **a** Structure of dioscin. **b** Cell viability analysis of A549 and H1299 cells **c** cultured in the presence of dioscin for 24 and 48 h by MTT assay. **d** A549 and H1299 cells were treated with different concentrations of dioscin (0–5 μM) for 24 h and then observed under microscopes to reveal cell death in a dose-dependent manner. Data represent mean of three determinations per condition repeated three times. Results are shown as mean ± SD

The chemical structure of dioscin was shown in Fig. 1a. To determine the cytotoxicity and the effect of cell proliferation of dioscin on A549 and H1299 cells, cells were treated with different concentrations of dioscin (0–5 μM) for 24 or 48 h. Results from MTT assay for cell viability revealed that dioscin inhibited growth in a dose-dependent and time-dependent manner (Fig. 1b, c). Furthermore, results from microscopic observation of cell morphology, as shown in Fig. 1d, indicated that significant cell death was observed at 24 h after dioscin treatment.

### Dioscin-induced cell apoptosis is dependent on the activation of caspase-3 and caspase-8 in human lung cancer cell lines

**Fig. 2:**
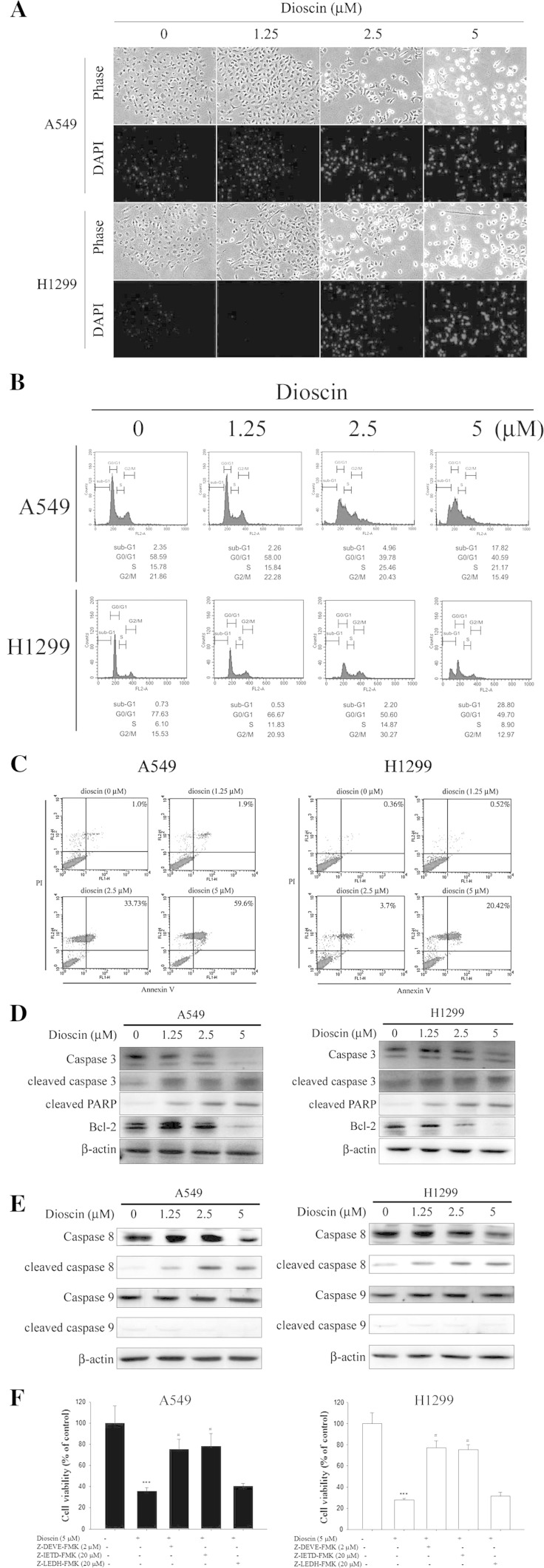
Dioscin-induced cell apoptosis in human lung cancer cell lines. **a** Cells were treated with different concentrations of dioscin (0–5 μM) for 24 h and then subjected to DAPI staining for DNA (*blue areas*) by fluorescence microscopy. **b** A549 and H1299 cells were incubated for 18 h in the absence of serum and then treated with indicated concentrations of dioscin for 24 h, after which the cells were stained with PI and analyzed for DNA content by flow cytometry. Furthermore, after being treated with different concentrations of dioscin for 24 h, cells were harvested and then subjected to quantitative analysis of cell apoptosis by annexin-V and PI double-stained flow cytometry (**c**), or subjected to Western blotting with an antibody against Bcl-2, PARP or caspase-3 antibody (**d**), as well as cleaved caspase-8 and caspase-9 (**e**). β-Actin acting as an internal control. **f** Cells were treated with 5 μM dioscin for 24 h in the presence or absence of 2 μM Z-DEVE-FMK, 20 μM Z-IETD-FMK and 20 μM Z-LEHD-FMK and then subjected to MTT assay to determine cell viability. Results are shown as mean ± SD. ***P < 0.001, control versus dioscin; #*P* < 0.05, dioscin versus Z-DEVE-FMK, Z-IETD-FMK and Z-LEHD-FMK plus dioscin

To determine whether the inhibitory effect of cell proliferation of dioscin is associated with induction of cell apoptosis, DAPI staining assay was performed to reveal the presence of nuclei condensing and apoptotic bodies in dioscin-treated A549 and H1299 cells in a dose-dependent manner (Fig. 2a). Further characterization analysis of the cell cycle by flow cytometry showed a dose-dependent increased accumulation of cell population in sub-G1 phase after a 24-h treatment with 2.5 and 5 μM of dioscin (Fig. 2b). Meanwhile, annexin-V and PI double-staining displayed an increased percentage of apoptotic cells after a 24-h treatment of dioscin of 2.5 and 5 μM (Fig. 2c), respectively. Furthermore, cleaved PARP and cleaved caspase-3 and caspase-8 were also detected in dioscin-treated A549 and H1299 cells, accompanied with decreased expression levels of Bcl-2 and anti-apoptosis protein **(**Fig. 2d, e). To further confirm the involvement of caspase activation in dioscin-induced apoptosis, caspase-specific inhibitors were employed to show that a pretreatment with caspase-3- or caspase-8-specific inhibitor could effectively attenuate dioscin-induced cell apoptosis, while caspase-9 inhibitor has no effect (Fig. 2f). These data suggested that dioscin-induced apoptosis is dependent on the activation of caspase-3 and caspase-8, but not that of caspase-9 in human lung cancer cell lines.

### Induction of autophagy in dioscin-treated human lung cancer cell lines

**Fig. 3:**
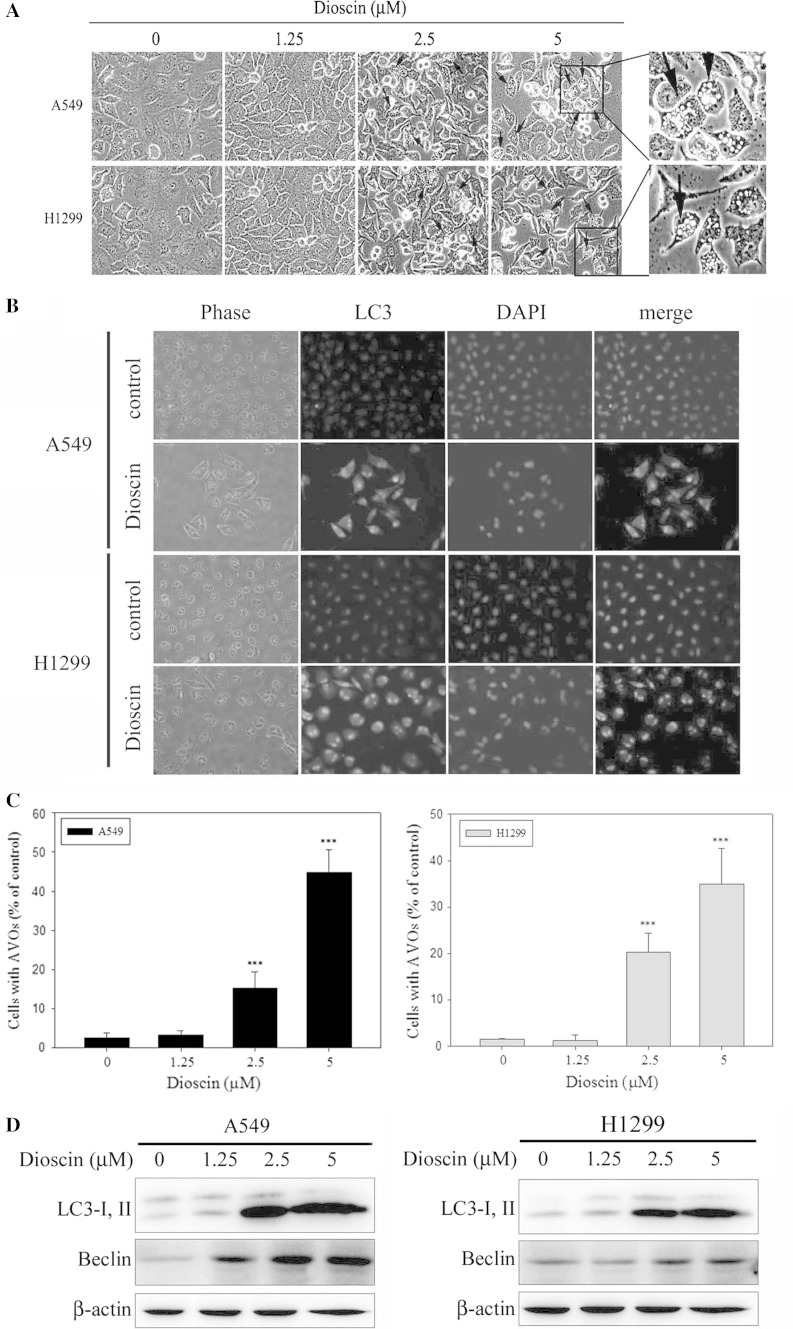
Induction of autophagy in dioscin-treated human lung cancer cell lines. **a** A549 and H1299 cells were treated with 1.25, 2.5 and 5 μM dioscin for 12 h and then observed under microscope to reveal the formation of various numbers of vacuoles in cell cytoplasm. **b** A549 and H1299 cells were treated with 5 μM dioscin for 12 h, followed by immunostaining and an observation of LC-3 (*green fluorescence*) and DAPI (*blue fluorescence*) under fluorescence microscopy. **c** The percentage of cells with the formation of AVOs was calculated based on the results of fluorescence-activated cell sorting assay. **d** Cells were treated with an indicated concentration of dioscin (0–5 μM) for 12 h and then subjected to Western blotting to study the expression levels of LC3-I, LC3-II and beclin-1 with β-actin acting as an internal control. Results are shown as mean ± SD. ***P < 0.001, control versus dioscin

Previous data showed that cells displayed characteristic apoptotic changes in cell morphology after 24-h treatment with dioscin. Moreover, various numbers of cytoplasmic vacuoles were observed at 12 h after dioscin treatment (Fig. 3a). Beclin-1, a Bcl-2-interacting protein, also binds to class III PI3Ks and helps to regulate autophagy. Therefore, the formation of LC3 puncta was analyzed by in situ immunostaining. As shown in Fig. 3b, cytoplasmic LC3 formation was observed in dioscin-treated A549 and H1299 cells, which indicated the formation of autophagosomes. A significant change in LC3 puncta formation was found at 12 h after dioscin treatment at a concentration of 5 μM. According to a previous study, autophagy is characterized by the formation of numerous acidic vesicles that are called AVOs (Zhan et al. 2012). Therefore, the next step is to assess whether dioscin may induce the formation of numerous AVOs in A549 and H1299. For quantification, cells with AVOs showed enhanced red fluorescence analyzed by flow cytometry, which significantly increased in a dose-dependent manner after treatment with dioscin (Fig. 3c). In addition, increased LC3-II and beclin-1 protein expression was observed in dioscin-treated A549 and H1299 cells, and such increase was in a dose-dependent manner (Fig. 3d).

### Dioscin-induced cell death was not rescued by treatment autophagy inhibitor

**Fig. 4:**
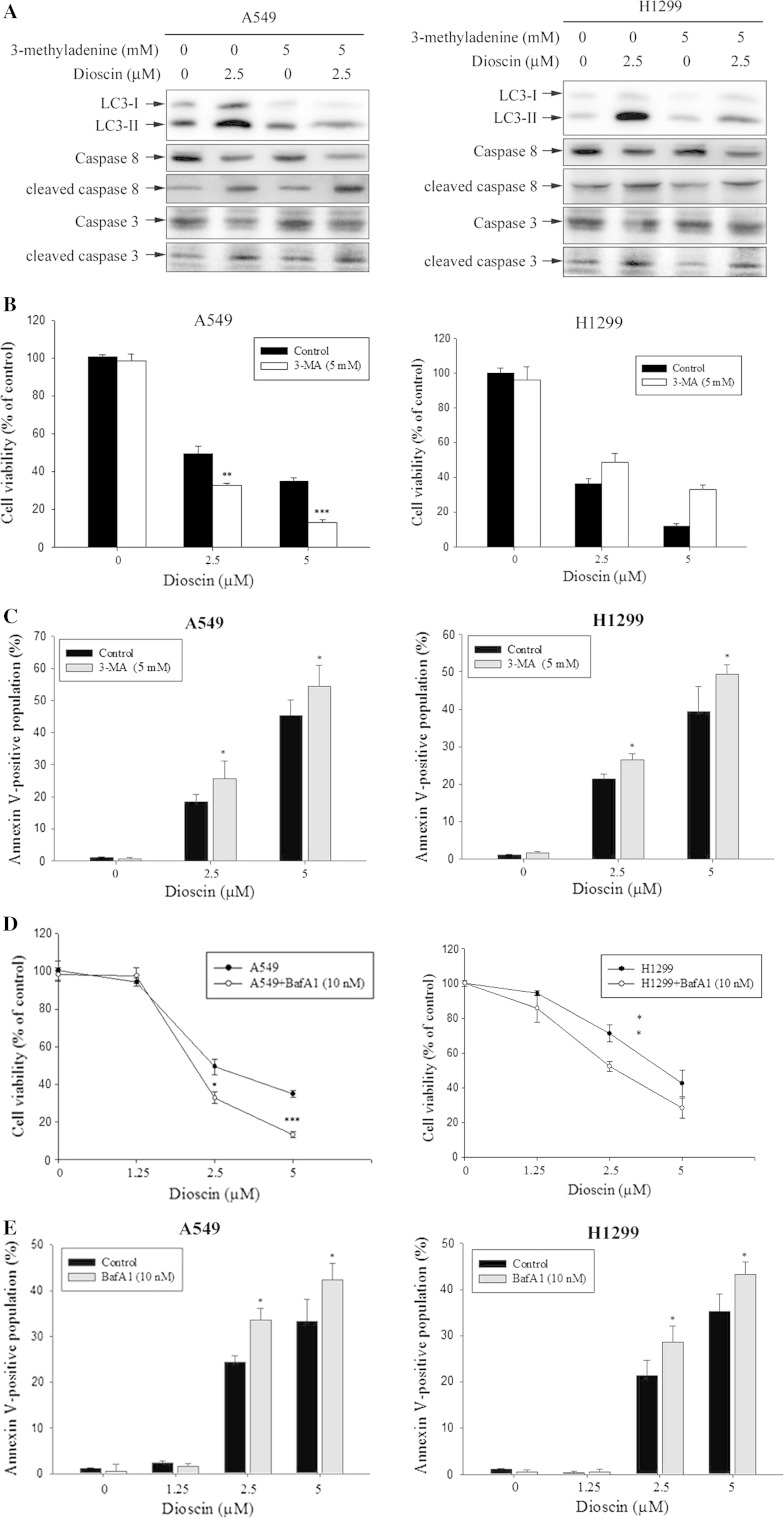
Effect of autophagy inhibitors on dioscin-induced human lung cancer cell death. A549 and H1299 cells were treated for 24 h with 5 μM dioscin with or without a 1 hour pretreatment of 5 mM 3-MA. The expression of LC3-II, cleaved caspase-3 and cleaved caspase-8 was detected by Western blotting (**a**), cell viability was determined by MTT assay (**b**), together with annexin-V and PI double-staining flow cytometry (**c**). Subsequently, A549 and H1299 cells were treated with different concentration of dioscin for 24 h with or without a pretreatment of 10 nM BafA1. Afterward, treated cells were subjected to MTT assay for cell viability (**d**), double-staining with annexin-V and PI and flow cytometry (**e**). Results are shown as mean ± SD. *P < 0.05, **P < 0.01, ***P < 0.001, dioscin versus 3-MA or BafA1 plus dioscin

In this part of study, 3-MA, an autophagy inhibitor by blocking autophagosome formation via the inhibition of type III PI-3K, was used for a pretreatment. As results shown in Fig. 4a, 1 h pretreatment with 3-MA of 5 mM before a dioscin treatment for 24 h led to a decreased level of dioscin-induced LC3-II protein, as well as increased levels of cleaved caspase-8 and cleaved caspase-3. 3-MA pretreatment also increased the inhibitory effect of dioscin on cell proliferation (Fig. 4b) while the percentage of annexin-V-positive cells was increased (Fig. 4c). Bafilomycin A1 (BafA1), an inhibitor of vacuolar ATPase and prevents the fusion between lysosomes and autophagosomes, was also used in the pretreatment. As shown in Fig. 4d, cells pretreated with BafA1 were more susceptible to dioscin (2.5 and 5 μM). A similar result was obtained in the annexin-V staining assay. Cells treated for 24 h with dioscin plus BafA1 showed an increased percentage of annexin-V-positive cells (Fig. 4e). Therefore, inhibition of autophagy could not inhibit dioscin-induced cell death, but enhanced dioscin-induced apoptosis.

### The autophagy induction by dioscin is dependent on a regulation of PI3K/Akt and MAPK signaling pathways in human lung cancer cell lines

**Fig. 5:**
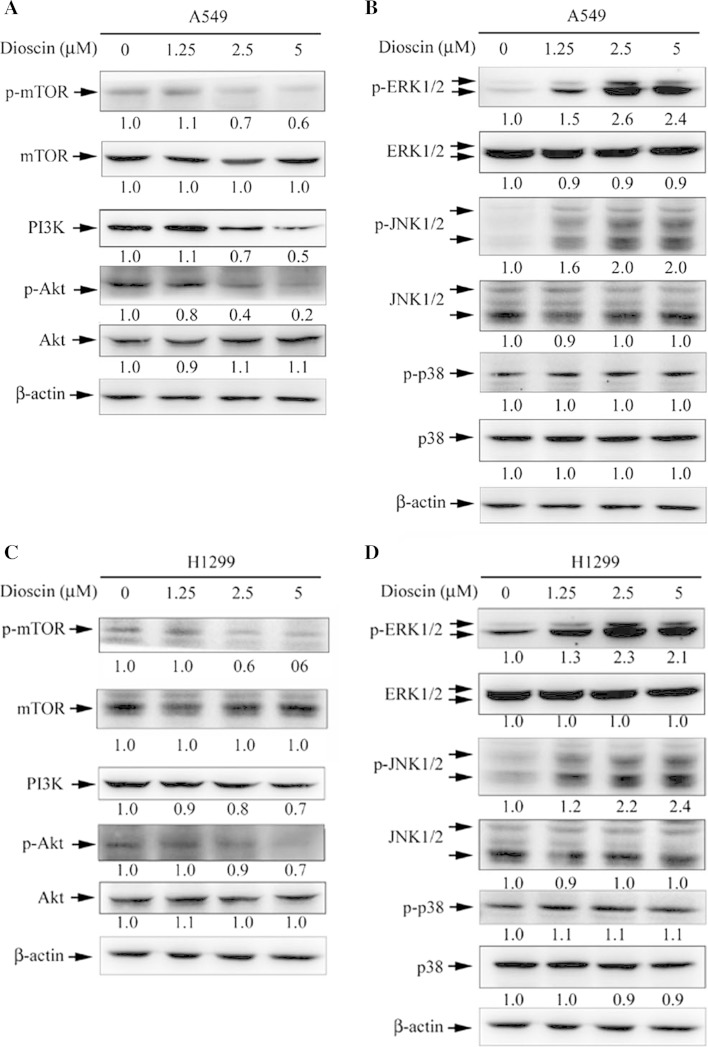
Dioscin inhibits the mTOR, PI3K and Akt and activates the ERK1/2 and JNK1/2 in human lung cancer cell lines. Cells were treated with different concentrations of dioscin (0–5 μM) for 24 h and then subjected to Western blotting with an antibody against phospho-mTOR, phospho-PI3K or phosphor-Akt (**a**, **c**). The levels of phosphorylation of ERK1/2, JNK1/2 and p38 were also investigated by Western blotting with β-actin being used as an internal control (**b**, **d**). The values under each lane indicate relative density of the band normalized to β-actin using a densitometer

Several signaling pathways have been implicated in the regulation of autophagy (Lee et al. 2008; Dong et al. 2002). In a further investigation into the underlying molecular mechanisms, the phosphorylation of mTOR was examined to show that dioscin treatment led to a decreased phosphorylation of mTOR (Fig. 5a, c). To investigate the possible role of MAPK pathways in dioscin-induced autophagy, the expression levels of PI3K and phosphorylated forms of Akt, ERK1/2, p38MAPK and JNK1/2 were examined by Western blotting. Results showed that the expression of PI3K and phosphorylation of Akt were decreased in cells treated with dioscin in a dose-dependent manner. On the contrary, dioscin led to an activation of ERK1/2 and JNK1/2 in a dose-dependent manner (Fig. 5b, d). The results showed that dioscin could induce autophagy by an up-regulation of ERK1/2 and JNK1/2 activation, as well as a down-regulation of PI3K/Akt and mTOR phosphorylation.

## Discussion

Natural herbal products have a promising and potential role in developing novel chemotherapeutics for various cancers (Casciola-Rosen et al. 1996; Lee et al. 2008). Dioscin has been extensively studied for its antitumor effect including antiproliferative activities, cell cycle arrest and apoptosis induction via the mitochondrial and some other pathways (Cai et al. 2002; Wang et al. 2007a, b; Sun et al. 2011). Cai et al. reported that after being treated with dioscin, caspase-9 and caspase-3 activity was increased in Hela cells, and the activity of caspase-8 did not change. However, another study showed dioscin-induced FasL and FADD expression, caspase-8 activation and Bid truncation in human myeloblast leukemia HL-60 cells. Results from our previous study performed on A549 and H1299 cells indicated that dioscin could significantly enhance the expression amounts of cleaved caspase-3, caspase-8 and PARP. These results suggested that the results of dioscin-induced caspase expression may have cell type-specific correlation. Meanwhile, the expression of the antiapoptotic protein Bcl-2 also was decreased. The findings are consistent with the apoptosis-inducing effects of dioscin on HeLa cells (Cai et al. 2002).

Autophagy is an important cellular response to numerous diseases. Recently, autophagy has become a potential and promising target in drug research for various diseases and also be implicated in the pathogenesis related to cancers and diseases (Shintani and Klionsky 2004; Kondo et al. 2005; Høyer-Hansen and Jäättelä 2008). Furthermore, many anticancer agents were reported to induce autophagy (Rosenfeldt and Ryan 2009). Sulforaphane causes autophagy as a defense mechanism against apoptosis in PC3 and LNCaP prostate cancer cells (Herman-Antosiewicz et al. 2006), while 7,7″-dimethoxyagastisflavone (DMGF) induced autophagic cell death in HepG2 cells (Hwang et al. 2012). The formation of vacuoles in dioscin-treated cells is similar to cell autophagy (Kitanaka and Kuchino 1999), a general phenomenon that occurs when cells respond to stress. Autophagy is a type II programmed cell death and a lysosomal degradation pathway essential for homeostasis (Kelekar 2005). When autophagy is induced, beclin-1 and LC3 distribute to the membrane of autophagosomes that are correlated to the extent of autophagosome formation (Kelekar 2005). In this study, dioscin induced autophagy as early as 12 h after the addition of dioscin, and results from the analysis of LC3-II expression indicated that the induction of autophagy was dose-dependent (Fig. 3).

Previous studies have suggested that autophagy can be induced by various compounds and involved in cell death or cytoprotection in HCC cells (Chua and Choo 2011). Autophagy is genetically programmed, and promoters of autophagy are clinically beneficial in the setting of cancer prevention. With autophagy inhibitors, 3-MA and BafA1, the role of autophagy in dioscin-induced cell death was further investigated. The dioscin-induced inhibition of LC3-II, as well as increase in procaspase-3 and procaspase-8 cleavage levels, and subsequently increased cell death were conversed by 3-MA (Fig. 4). Therefore, we hypothesized that an inhibition of autophagy could not decrease, but rather enhance, dioscin-induced cell death. Furthermore, a treatment with caspase inhibitor alone led to a limited recovery of cell viability (Fig. 2f), suggesting that autophagy serves as a critical defensive mechanism against common chemotherapeutic agents in A549 and H1299 cell lines.

For a better understanding of dioscin-induced cytotoxicity, the downstream effects of dioscin were further defined. As autophagy is required for the effective management of metabolic stress, promoting autophagy through mTOR pathway inhibition is reasonably expected to limit tumor progression (Rimando et al. 2004). The PI3K/Akt and mTOR/p70S6K pathways are main pathways that negatively regulate autophagy (Suh et al. 2007). This study demonstrated that the mechanism of dioscin-induced autophagy of A549 and H1299 cells through an inhibition of PI3K/Akt/mTOR pathways and activation of ERK1/2 and JNK1/2 signal pathway (Fig. 5).

In conclusion, through this study, we demonstrated that dioscin suppressed cell growth and induced apoptosis in A549 and H1299 cells. Autophagy has been regarded as a double-edged sword in cancer development, progression and responses to treatment (Lozy and Karantza 2012). Previous studies showed that an induction of autophagy appears to facilitate therapy-induced killing of tumor cells (Longo et al. 2008; Ko et al. 2009). Otherwise, the later has been shown to be due to its role in generating glycolytic substrates through recycling damaged organelles and mutant and unfolded proteins, thereby providing survival advantage to cancer cells under nutrition starvation and cellular stress (Lozy and Karantza 2012). In our study, dioscin induced autophagy in the early stage of dioscin-induced apoptosis, leading to a suggestion that autophagy protects cancer cells from the anticancer activity of dioscin. We also found that dioscin induced autophagy through an inhibition of the PI3K/Akt/mTOR and an activation of ERK1/2 and JNK1/2 signal pathway. With the ability to induce apoptotic and autophagic effects, dioscin has promising anticancer properties in human lung cell lines. Therefore, dioscin may act as a new and potential anticancer agent for human lung cancer cell lines.
